# Insights Into the Low Rate of In-Pump Thrombosis With the HeartMate 3: Does the Artificial Pulse Improve Washout?

**DOI:** 10.3389/fcvm.2022.775780

**Published:** 2022-03-11

**Authors:** Peng Fang, Jianjun Du, Andrea Boraschi, Silvia Bozzi, Alberto Redaelli, Marianne Schmid Daners, Vartan Kurtcuoglu, Filippo Consolo, Diane de Zélicourt

**Affiliations:** ^1^School of Mechanical Engineering and Automation, Harbin Institute of Technology, Shenzhen, Shenzhen, China; ^2^The Interface Group, Institute of Physiology, University of Zurich, Zurich, Switzerland; ^3^Department of Electronics, Information and Bioengineering, Politecnico di Milano, Milano, Italy; ^4^Product Development Group Zurich, Department of Mechanical and Process Engineering, ETH Zurich, Zurich, Switzerland; ^5^Anesthesia and Intensive Care, IRCCS San Raffaele Scientific Institute, Milano, Italy; ^6^Università Vita Salute San Raffaele, Milano, Italy

**Keywords:** left ventricular assist device (LVAD), computational fluid dynamics (CFD), HeartMate 3 (HM3), pump thrombosis, washout, rotational speed modulation, wall shear stress (WSS)

## Abstract

While earlier studies reported no relevant effect of the HeartMate 3 (HM3) artificial pulse (AP) on bulk pump washout, its effect on regions with prolonged residence times remains unexplored. Using numerical simulations, we compared pump washout in the HM3 with and without AP with a focus on the clearance of the last 5% of the pump volume. Results were examined in terms of flush-volume (*V*_*f*_, number of times the pump was flushed with new blood) to probe the effect of the AP independent of changing flow rate. Irrespective of the flow condition, the HM3 washout scaled linearly with flush volume up to 70% washout and slowed down for the last 30%. Flush volumes needed to washout 95% of the pump were comparable with and without the AP (1.3–1.4 *V*_*f*_), while 99% washout required 2.1–2.2 *V*_*f*_ with the AP vs. 2.5 *V*_*f*_ without the AP. The AP enhanced washout of the bend relief and near-wall regions. It also transiently shifted or eliminated stagnation regions and led to rapid wall shear stress fluctuations below the rotor and in the secondary flow path. Our results suggest potential benefits of the AP for clearance of fluid regions that might elicit in-pump thrombosis and provide possible mechanistic rationale behind clinical data showing very low rate of in-pump thrombosis with the HM3. Further optimization of the AP sequence is warranted to balance washout efficacy while limiting blood damage.

## Introduction

Heart failure is a rapidly expanding and lethal cardiovascular disease with a 30% 1-year mortality rate in older adults (1). Left ventricular assist devices (LVADs) offer a lifesaving option for patients with advanced heart failure, restoring the cardiac output their heart can no longer provide (2). Over the past decades, LVADs have evolved from bridge-to-transplant to destination therapy and have become a viable alternative to heart transplantation with comparable outcomes (3–5). However, patients with LVAD still suffer from high complication rates, including thrombosis, bleeding, and strokes (4), which have at least in part been attributed to the hemodynamics of the implanted pump (6–8).

The HeartMate 3 (HM3) (Abbott, Chicago, Illinois, USA) has attracted attention due to its remarkably low rate of in-pump thrombosis (0 and 1.1% at 6 and 24 months after implantation, respectively) (9) compared to earlier devices such as the HeartMate II (HMII) (Abbott, Chicago, Illinois, USA) or to the HeartWare ventricular assist device (HVAD) (Medtronic, Minneapolis, Minnesota, USA) that showed a 10.7 and 6.4% rate of pump exchange due to pump thrombosis at 2 years, respectively (10). Distinctive features of the HM3 include a fully magnetically levitated rotor, large secondary flow paths, and its operative regime with the artificial pulse (AP) rotor speed modulation sequence (11–13). With a 2,000-rpm decrease in rotor speed followed by a 4,000-rpm increase before returning to baseline within the span of 350 ms, the AP entails a large and rapid change in rotor speed with the set goal to improve washout (11, 13). Accordingly, potential beneficial effects of the AP in terms of pump thrombogenicity have been suggested (14). However, whether the AP plays a role in the very low rate of clinically observed in-pump thrombosis or whether these may be solely due to the pump geometrical features remains unclear.

Previous studies assessing the impact of rotor speed modulation sequences have suggested that these may enhance arterial pulsatility via transient loading of the left ventricle (15). In the registry to evaluate the heartWare left ventricular assist system (ReVOLVE) study, patients with HVAD with the Lavare Cycle enabled showed significantly lower rates of strokes than those without, but the rate of in-pump thrombosis was unchanged (16). In line with these observations, experimental and computational fluid dynamics (CFD) studies suggested that, while the Lavare Cycle might improve ventricular washout (16), it did not have a significant impact on the HVAD flow fields or pump washout (17). As the AP is an inherent component of the HM3 system and cannot be switched off, isolated impact of the AP cannot be assessed clinically, requiring *in-vitro* or *in-silico* approaches. Our earlier study in the area suggested that under the tested conditions, the AP did not improve overall pump washout, defined as the time required for 95% of the blood present in the HM3 at a given time point to exit the pump (17, 18). Washout of the remaining 5% was not in the scope of those studies. However, in terms of in-pump thrombosis, understanding the distribution and clearance rate of the last 5% of so-called “old blood” remaining in the pump is of prime interest, as these 5% are the ones with the longest residence time. While it may be inferred that by alternating flow disturbances (which may increase mixing) and sudden acceleration (which may then increase washout), the AP may help in the clearance of otherwise hard-to-clear fluid regions; no study has investigated its actual contribution to improving internal pump hemodynamics and limiting the risk of in-pump thrombosis. Furthermore, because the AP duration (0.35 s) is on the same order as the characteristic 95% washout time (~0.3 s at 5 L/min) (17), a time-dependent understanding of the HM3 washout pattern is warranted.

In this study, we conduct a comprehensive investigation of the effect of the AP on the washout in the HM3, with a focus on slow washout regions as a surrogate marker of elevated risk of in-pump thrombus formation. In detail, we analyze wall shear stress (WSS) patterns, regions of flow stasis, clearance times, and clearance patterns for five different reference time points, providing a better understanding of the effect of the AP on bulk and local washout. Results are analyzed in terms of both the washout time and nondimensional flush volume, defined as number of times the pump was flushed with new blood, providing insights into the effect of the AP and associated rotor acceleration and deceleration independent of the pump flow rate.

## Materials and Methods

### Computational Methods

**Figure 1:**
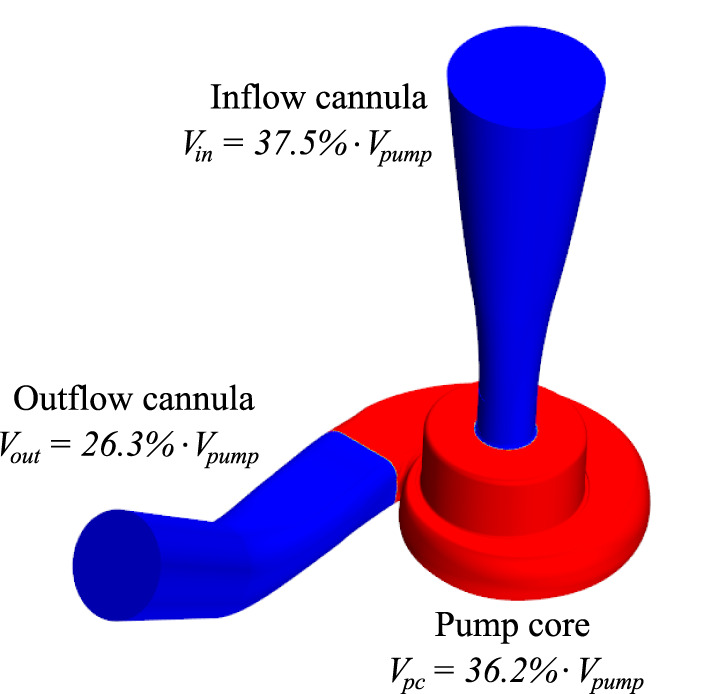
Visualization of the domain splitting retained for analysis by parts. *V*_*pump*_ is the pump volume and *V*_*in*_, *V*_*pc*_, and *V*_*out*_ are the volumes of the inflow cannula, pump core, and outflow cannula, respectively.

Numerical simulations of the blood flow within the HM3 with and without the AP were conducted in STAR-CCM+ (Siemens, Munich, Germany). The HM3 pump geometry and mesh were obtained from Boraschi et al. (17). The mesh contained 10 million polyhedral grid elements, including an 8-element boundary layer along the rotor, a 10-element boundary layer along the stator, and local mesh refinement where needed. Unsteady Reynolds-averaged Navier–Stokes simulations were conducted using implicit second-order temporal and spatial discretization, Menter's Shear Stress Transport (SST) k-ω turbulent modeling, dynamic time-stepping (programmed to correspond to 2° of rotation per time step), and a 10^−5^ convergence criterion for the residual error. Blood was considered as an incompressible Newtonian fluid (ρ = 1, 050 *kg*/*m*^3^ and μ = 3.5 *mPa*·*s*). For regional analysis, the pump fluid volume was divided into three parts: the inflow cannula, the pump core, and the outflow cannula (Figure 1). More detailed information on the HM3 geometry, grid independence tests, validation, and computational methods can be found in our previous studies (17, 18).

### Simulation Scenarios and Boundary Conditions

**Figure 2:**
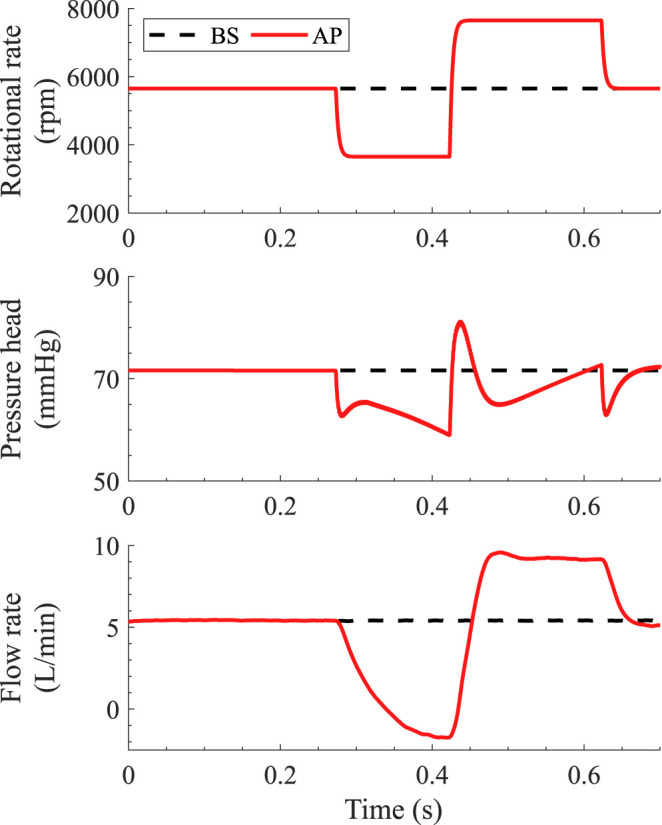
Pump rotational speed **(A)**, pump pressure head **(B)** and pump flow rate **(C)** during the artificial pulse and under baseline conditions. The pump pressure head associated with the different conditions was obtained from the lumped parameter heart-pump interaction model as described in Simulation Scenarios and Boundary Conditions. Rotational speed and pressure head are imposed as boundary conditions in the computational fluid dynamics (CFD) simulations, while the pump flow rate is a result of the CFD simulations.

To isolate the impact of the AP, we compared the results obtained with the AP virtually turned on or off. For both the conditions, inlet and outlet pressure boundary conditions were obtained from a lumped parameter model (19) representing the cardiovascular system of an average patient with LVAD—with a heart rate of 91 bpm, end-diastolic left ventricular volume of 345 ml, and ejection fraction of 10.3% prior to LVAD implantation (20), implanted with the HM3 LVAD. Dynamic hydraulic properties of the HM3 were derived from (21). To assess the effect of the AP in isolation, residual left ventricular pulsatility was turned off for these simulations and the baseline set-speed of the AP defined according to (17, 18) aiming to achieve a clinically relevant mean pump flow of 5 L/min over the 0.35 s of the AP duration. The resulting rotational speed sequence for the AP scenario entailed a baseline set-speed of 5,650 rpm, a low-speed phase at 3,650 rpm for 0.15 s, and a high-speed phase at 7,650 rpm for 0.2 s (Figure 2A). The pump pressure head varied between 59.0 and 81.2 mm Hg over the AP cycle (Figure 2B). Time-dependent rotational speed and pump pressure head derived from the lumped parameter model were imposed as boundary conditions for the CFD simulations. In the baseline scenario with the AP virtually turned off, the pump was operated at the same rotational speed and pressure as during the baseline conditions of the AP scenario, namely, 5,650 rpm and 71.6 mm Hg, respectively.

Finally, we further simulated a low-flow condition (3,600 rpm, 3.5 L/min) and a high-flow condition (7,600 rpm, 8.6 L/min) to assess the dependence of the baseline washout on the pump flow rate and rotational speed.

### Data Analysis

#### Volume Washout

To investigate volume washout, we quantified the clearance of a passive “old blood” scalar (OBS) by pure advection:


(1) $$\begin{array}{c} \rho \frac{\partial [OBS](\vec{x},t)}{\partial t}\;+\rho \nabla \cdot (\vec{u}\cdot [OBS])\;=\;for\;t\;>\;t_{i} \end{array}$$

where *t* is time, $\vec{x}$ is the position vector, [*OBS*] is the OBS concentration, ρ is the blood density, $\vec{u}$ is the blood velocity, and *t*_*i*_ is the OBS scalar initialization time. The initial scalar concentration, [*OBS*]_0_, was set to 100% and [*OBS*] is set to zero at the inflow. The 7 cm extension of the outlet allows for adequate handling of reverse flow during the AP without influence of the outlet boundary condition on the OBS concentration within the pump. For the AP, we studied the washout of five OBS (denoted AP1 through AP5) with initialization times chosen such that the residual fraction of OBS was around 5, 10, 25, 50, and 100% at the beginning of the AP, respectively.

#### Residual Fraction of OBS

We define the OBS residual fraction, *rOBS*, as:


(2) $$\begin{array}{r} rOBS\left(t\right)\;=\;\frac{1}{V\cdot {\left[OBS\right]}_{0}}\cdot \int_{V}\left[OBS\right]\left(t\right)\cdot \partial \;V \end{array}$$

where *V* is the integration volume. Unless otherwise specified, rOBS refers to the residual OBS concentration in the whole pump.

#### Flush Volume

To assess whether the differences in pump washout solely relate to differences in pump flow rate or whether the acceleration and deceleration imparted by the AP provide further advantages, we define the nondimensional flush volume, *V*_*f*_, associated with each OBS as:


(3) $$\begin{array}{r} V_{f}\left(t,t_{i}\right)\;=\;\frac{1}{V_{pump}}\int_{t_{i}}^{t}Q\left(\tau \right)\partial \tau \end{array}$$

where *t*_*i*_ is the OBS initialization time, *V*_*pump*_ is the pump volume, and *Q* is the signed pump flow rate. *Q* is positive when new blood flows from the ventricle toward the aorta and Q is negative when the flow reverses and old blood flows back from the outlet graft into the pump. *V*_*f*_ describes the number of times the pump was flushed with new blood since OBS initialization.

#### Reference OBS Residual Fraction

To provide a reference for pump washout rates, we define for each OBS a reference OBS residual fraction for the whole pump, *rOBS*_*ref*_, assuming perfect flushing of the old blood by the new one:


(4) $$\begin{array}{r} rOBS_{ref}\left(t,t_{i}\right)\;=\;1\;-\;V_{f}\left(t,t_{i}\right) \end{array}$$

With the above definition *rOBS*_*ref*_ = 0% when *V*_*f*_ = 1, i.e., the old blood would be entirely washed out after flushing the pump with once its volume of new blood.

#### Wall Shear Stress

Finally, to evaluate the effect of the AP on surface washout, we compared WSS patterns across conditions. We monitored instantaneous WSS variation at selected locations in area of flow stagnation along the top and bottom of the casing (see Supplementary Figure S1 for the location of the monitoring points).

## Results

### Overall old Blood Scalar Washout With and Without AP

**Figure 3:**
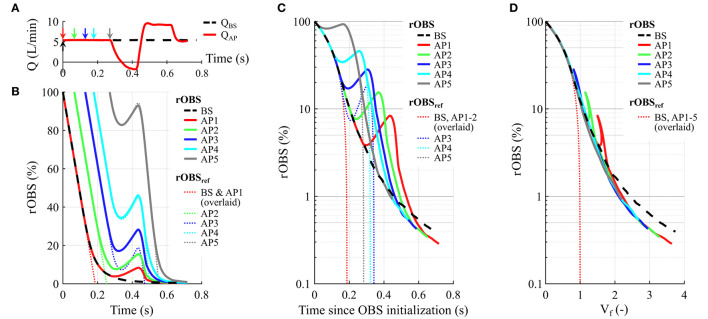
Effect of the artificial pulse (AP) on the residual fraction of old blood scalar (rOBS). **(A)** Pump flow rate under baseline condition (BS) and with the AP. Arrows illustrate the different OBS initialization times using the same color coding as in **(B–D)**. Specifically, we consider one OBS for baseline (BS initialized in *t* = 0, black dashed arrow) and five different OBS initialization times with the AP (AP1–5, full colored arrows). **(B)** rOBS as a function of time (thick lines). For each OBS, the thin dotted lines illustrate the temporal evolution of the reference residual OBS concentration, rOBS_ref_, providing a reference for the best possible washout given the pump flow rate. **(C)** rOBS as a function of time since initialization. A logarithmic scale is used to visualize differences for rOBS below 5%. 99% washout times for BS and AP3–5 are comparable despite the AP flow reversal and reduced flush volume for AP3–5 as illustrated by the longer rOBS_ref_ clearance times (thin dotted lines). **(D)** rOBS as a function of the flush volume, *V*_*f*_. Per definition, all the rOBS_ref_ curves are overlaid depicting the best case scenario where one flush volume suffices to clear the pump. The “shark fin” feature corresponds to the AP flow reversal. The rotor acceleration and high-speed phase accelerate OBS washout for an equivalent flush volume as illustrated by the faster decline of rOBS for AP1–5 compared to baseline. The rOBS time course for all the simulated scenario, including high- and low-flow conditions, is given in Supplementary Material S3.

For all the tested conditions, the residual OBS concentrations, rOBS, initially closely follow the associated reference curves, *rOBS*_*ref*_ (Figure 3B). The difference between simulated and reference residual concentrations is below 0.001% down to rOBS = 50% for baseline and AP1–4 and below 2% down to rOBS = 30% for all the tested conditions (Supplementary Material S2). For the first 70% of the pump volume, OBS clearance initially scales almost one-to-one with the overall flush volume, so that all the curves initially collapse onto one another when plotted against *V*_*f*_ (Figure 3D). The same pattern is observed under the high- and low-flow conditions (Supplementary Material S3).

Under the simulated conditions, the low-speed phase of the AP leads to a transient flow reversal (Figure 3A) associated with an increase in all the rOBS (Figure 3B, AP1–5). This translates into a “shark fin” feature in Figure 3D because the net *V*_*f*_, decreases for negative pump flow rates, reflecting backflow of old blood from the outflow graft into the pump. Flow reversal does not lead to a “shark fin” feature for rOBS_AP4_ and rOBS_AP5_, as the curves follow the reference washout curve, *rOBS*_*ref*_, during that phase.

Taken together, the alternating low- and high-speed phases of the AP translate into prolonged 95% washout times compared to baseline (Figure 3C and Table 1). However, depending on the initialization time considered, the mean pump flow rate during the washout period changes as well, decreasing from 5.42 L/min at baseline to 4.94 L/min for AP5 and 4.25 L/min for AP1 (Table 1). Accounting for the effect of flow rate, flush volumes required to reach 95% washout are comparable across conditions, ranging between 1.3 and 1.4, with the exception of AP1 for which the AP started just before the retained rOBS = 5% assessment cutoff (rOBS_AP1_ = 5.9% at AP start, Table 1). In contrast, the number of flush volumes required to reach 99% washout decreases from 2.5 at baseline to 2.1–2.2 with the AP or under high-flow condition.

**Table 1:** 95 and 99% washout times for different old blood scalars considered in the study.

**Old blood scalar**	**rOBS at AP start (%)**	**95% washout time**		**99% washout time**		
		**Time (s)**	***V_***f***_*** **(–)**	**Time (s)**	***V_***f***_*** **(–)**	**$\bar{Q_{99\%}}$ (L/min)**
Baseline	–	**0.26**	1.4	0.46	2.5	5.42
AP1	5.9	0.26 (1st), 0.47 (2nd)	1.4 (1st), 1.7 (2nd)	0.53	2.2	4.25
AP2	11.9	0.41	1.4	0.49	2.1	4.39
AP3	24.5	0.38	1.4	0.46	2.1	4.60
AP4	56.9	0.36	1.4	0.45	2.1	4.84
AP5	100.0	0.31	**1.3**	**0.44**	2.1	4.94
Low flow	–	0.41	1.3	0.72	2.4	3.50
High flow	–	0.17	1.3	0.27	2.1	8.60

*Minimum washout times are marked in bold*.

*$\bar{Q_{99\%}}$: Time-average of the pump flow rate between OBS injection time and the 99% washout time*.

### Clearance Modes

**Figure 4:**
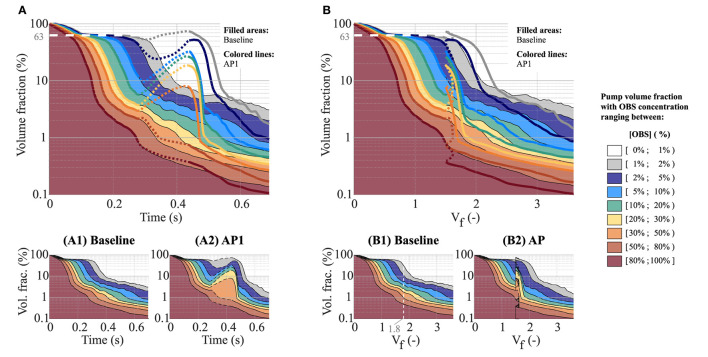
Effect of the AP on the fraction of the pump volume with high to low OBS concentrations as a function of time **(A,A1,A2)** and flush volume **(B,B1,B2)**. **(A1,A2,B1,B2)** show the results for baseline and AP1 individually. The dashed contour lines highlight the low-speed phase of the AP, which, due to the associated flow reversal, leads to a transient decrease in the net flush volume, *V*_*f*_ **(B2)**. **(A,B)** To ease the comparison between baseline and AP1, **(A,B)** show the superposition of **(A1,A2,B1,B2**), respectively. The colored areas depict the results under baseline conditions [corresponding to **(A1,B1)**, respectively], while the thick colored lines correspond to AP1 [corresponding to **(A2,B2)**, respectively]. Here again, the dashed lines highlight the low-speed phase of the AP. Note that, for all the panels, a logarithmic scale is used for the Y-axis.

Looking at the distribution of regions with high to low OBS concentration as washout progresses (Figure 4), different phases can be identified. At first, the sum of fluid volumes with ≥1% OBS decreases rapidly. The pump volume can be broadly categorized into regions with >80% OBS and regions with <1% OBS (dark red and white areas in Figure 4), suggesting that old blood is replaced almost one-to-one by new blood in the main flow paths. In the second phase, the decline in rOBS given in Figure 3 is no longer associated with a net increase in regions with new blood only ([OBS] <1%, white areas), but rather by mixing of old and new blood within the pump core and the outlet downstream. At baseline, the sum of fluid volumes with [OBS] > 1% remains relatively stable between *V*_*f*_ = 0.55 and *V*_*f*_ = 1.4 only decreasing from 66 to 60%, corresponding approximately to the combined pump core and outlet volumes (63%, horizontal white line in Figures 4A,B). Mixing phases between old and new blood are manifest by the appearance of fluid fractions with intermediate concentrations. The sum of fluid regions with [OBS] > 1% declines steeply after the residual OBS concentration in the core pump drops below 1% (*V*_*f*_ = 1.8, Figures 4B1, 5B1) and mixed blood is pushed toward the outlet. The same is observed for higher concentration bands. For example, the sum of fluid volumes with [OBS] > 50% declines steeply after the residual OBS concentration in the core pump drops below 50% (*V*_*f*_ = 0.56). The last phase is characterized by a drastic reduction of the clearance rate of all the concentration bands, testifying for the presence of fluid regions with prolonged residence times. While the overall rOBS is reduced to 5% with 1.4 flush volumes at baseline (Table 1) with only 4% of the pump volume having OBS concentrations higher than 20% (Figure 4B), it takes an additional 1.1 flush volumes to reduce both of these to 1%.

**Figure 5:**
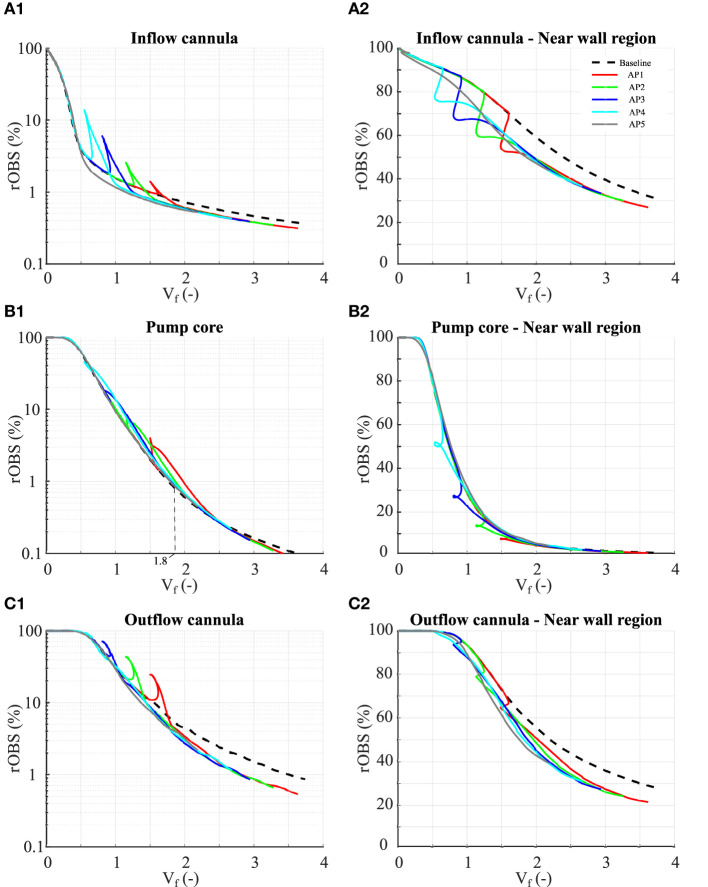
Residual OBS concentrations in the inflow cannula **(A1–A2)**, pump core **(B1–B2)**, and outflow cannula **(C1–C2)**. Definition of the different parts is given in **Figure 1. (A1–C1)** Residual OBS concentrations for the each pump part. Note that the results are plotted using a logarithmic scale for the residual OBS concentrations. **(A2–C2)** Residual OBS concentration in the vicinity of the pump surfaces (≤ 40 μm away from the walls). In total, these near-wall regions occupy 0.92% of the whole pump volume, 0.62% of the inlet, 1.44% of the pump core, and 0.71% of the outlet volumes.

Due to the flow reversal, the low-speed phase of the AP (illustrated by dotted lines in Figure 4) leads to backflow of mixed old and new blood back from the outflow graft into the pump illustrated by the increase in fluid volumes containing low to medium OBS concentrations (1 ≤ [OBS] ≤ 50%). The flow reversal still benefits the clearance of regions with high OBS concentrations with a continuous decline of the pump volume with [OBS] ≥ 50% (Figure 4B). The high-speed phase leads to a faster and deeper reduction of regions of high OBS concentration than at baseline. The sum of the regions with more than 20% OBS accounts for <1% of the pump volume already at *V*_*f*_ = 1.85 for AP1 vs. 2.5 for baseline (Figure 4B).

### Old Blood Scalar Clearance per Part

Figure 5 shows the OBS washout for the three pump parts individually (see Supplementary Figures 4, 5 for the complete set of results including the flow sensitivity study). The residual OBS concentration in the inflow cannula initially decays linearly, as only little mixing occurs in that part, and drops below 1% within 1 flush volume (Figure 5A1). OBS in the near-wall regions (here defined as <40 μm away from the walls) accounts for the majority of the OBS left in the inflow cannula after *V*_*f*_ = 1 (Figure 5A2) and is only slowly washed out. In the pump core, the residual OBS concentration follows an exponential decay, consistent with mixing, reaching 99% clearance within 1.5 flush volumes from the point at which new blood reached the pump core (Figure 5B1). Remaining OBS in the pump core is below 0.1% by *V*_*f*_ = 3.5, with good clearance of the near-wall regions (Figure 5B2). OBS clearance is the slowest in the outflow cannula, requiring 3.1 flush volumes from the point at which new blood reached the outlet to drop below 1% at baseline (Figure 5C1). The OBS secluded in the near-wall regions (Figure 5C2) accounts for <10% of the OBS remaining in the outflow cannula after *V*_*f*_ = 2.0, suggesting other slow clearance regions.

Compared to baseline, the AP leads to a transient increase in rOBS in the different pump parts followed by a faster and deeper OBS clearance during the acceleration phase (leading to the aforementioned “shark fin” feature, Figures 5A1–C1). By reverting flow direction along the walls, flow reversal favors clearance of the near-wall regions consistent with the decay of high volume with high OBS concentrations noted in Figure 4. The cumulative effect of the low- and high-seed phases is negligible in the pump core and strongest in the outflow cannula. After *V*_*f*_ = 2.0, rOBS in the outflow cannula is 1–1.5% lower with the AP than at baseline (Figure 5C1) and rOBS values in the near-wall regions of the inflow and outflow cannula are 5–12% lower than at baseline (Figures 5A2,C2). Improved near-wall washout accounts for more than 50% of the benefit observed in the inflow cannula, but only for 10–20% of the benefit noted in the outflow cannula.

### Washout of the Secondary Flow Paths of the Pump Core

**Figure 6:**
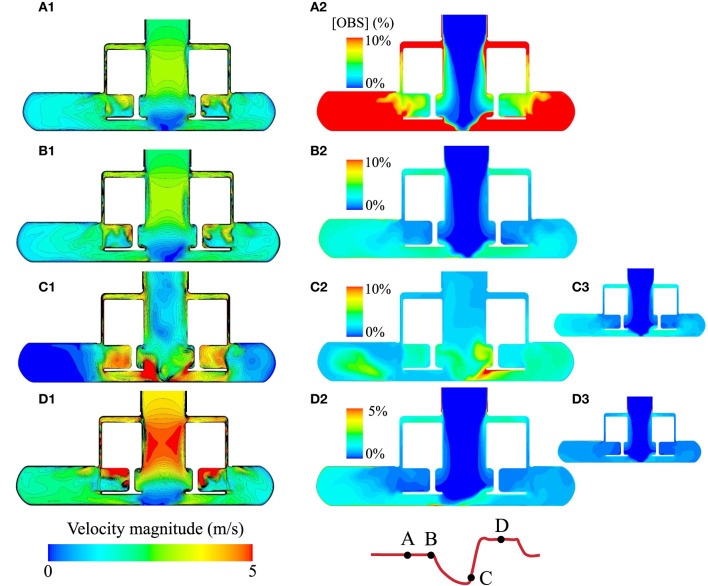
Comparison of the instantaneous velocity fields and evolution of the OBS concentrations in the pump core during the AP1 scenario. **(A1–D1)** Instantaneous velocity fields in the HM3 pump core for the four instants illustrated on the flow curve with the pump operating at baseline speed (5,650 rpm, instants A and B), low speed (3,650 rpm, instant C), and high speed (7,650 rpm, instant D). For AP1, the time points A, B, C, and D correspond to *V*_*f*_ = 0.9, 1.4, 1.5, and 2.0, respectively. **(A2–D2)** OBS concentrations at instants A, B, C, and D for the AP1 scenario. OBS concentrations are expressed as a percentage of the initial concentration [OBS]_0_. rOBS over the core pump for the AP1 scenario is 14.0, 2.6, 4.0, and 0.9% at the instants A, B, C, and D, respectively. **(C3,D3)** OBS concentrations in the pump core for *V*_*f*_ = 1.5 and 2.0 under baseline conditions, corresponding to the same flush volumes as instants C and D for AP1. Note that instants A and B apply to the baseline scenario as well, as the pump is operating under the set baseline speed at those time points.

As also noted in previous publications (17, 18), a region of low velocity forms centrally below the rotor during forward flow (Figures 6A1,B1,D1). The low-speed phase of the AP eliminates this low velocity region (Figure 6C1). The overall increase in OBS caused by the flow reversal is quickly counteracted during the high-speed phase (Figure 6D2). However, it is noteworthy that this flow stagnation region is unstable even at baseline (with a spatial shift between A1 and B1 for example) and, thereby, not associated with prolonged OBS residence times.

**Figure 7:**
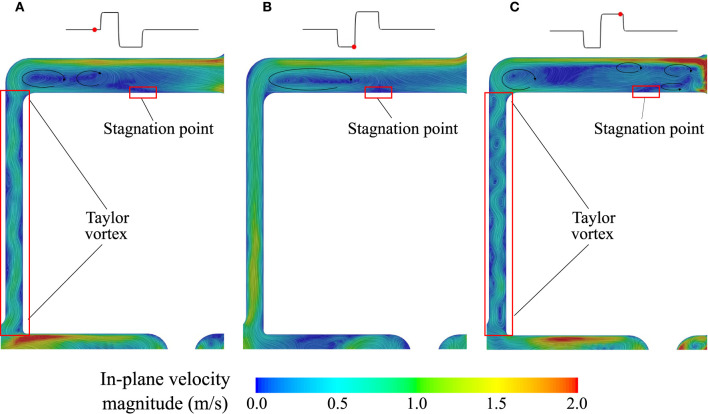
Velocity field in the secondary flow path. **(A)** Baseline, **(B)** Low-speed phase of the AP, and **(C)** High-speed phase of the AP.

In the secondary flow path (Figure 7), the net flow direction is from the outer casing toward the center. A stagnation point forms on the upper surface of the rotor where a delicate balance between centrifugal force and pressure difference is achieved. Modulation of the rotor speed induces a spatial shift of that stagnation point. The formation of Taylor–Couette–Poiseuille flow pattern, common in rotating machinery (22, 23), along the outer casing, is also of note. Their spatial frequency is directly modulated by the rotor velocity.

**Figure 8:**
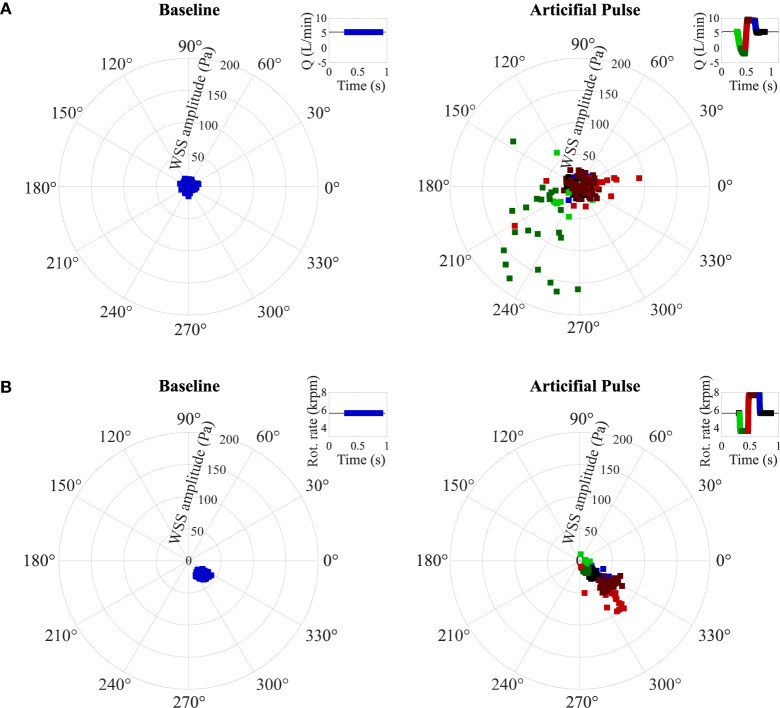
Wall shear stress on the central points of **(A)** the bottom of casing and **(B)** the top of casing within secondary flow path. Five outliers in **(A)** (with WSS higher than 200 Pa) are not shown. WSS: wall shear stress (see Supplementary Material S1 for the location of the WSS monitoring points).

We monitored WSS close to the two above noted stagnation regions (Figure 8). Centrally under the rotor, WSS oscillates between 0.19 and 15.57 Pa at baseline, in line with the unstable stagnation point location noted above. These WSS increase over 100 Pa (maximum 576.20 Pa) during the flow reversal (Figure 6C1) and return close to their baseline value during the high-speed phase of the AP because the stagnation region remains in the same location as at baseline. In the mid-radial location of the upper surface of the rotor, maximum WSS reaches 41.49 Pa at baseline and vary from 8.63 to 100.50 Pa during the AP deceleration and acceleration phases. For both the baseline and AP, the WSS direction is mainly concentrated between 300 and 330°.

### Effect of the AP on Outlet Clearance

**Figure 9:**
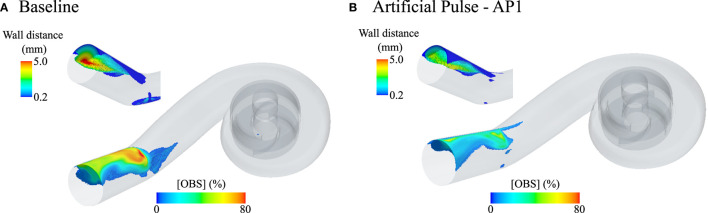
Fluid volumes in the HeartMate 3 (HM3) pump core and outlet cannula with residual OBS concentrations over 5% after flushing the pump with two times its blood volume (*V*_*f*_ = 2.0) without AP [**(A)**, baseline conditions] and with AP [**(B)**, AP1 scenario]. At *V*_*f*_ = 2.0, the average residual OBS concentration is 1.7 and 1.5% in the core pump for the Baseline and AP1 scenario, respectively, and is 4.5% at baseline and 3.5% for AP1 in the outlet cannula. For ease of visualization, only fluid volumes with [OBS] ≥ 5% and more than 200 μm away from the pump surfaces are shown. Apart from a small region under the rotor at baseline, no 5% OBS isovolume are identified in the pump core at that instance. The insets show the 5% OBS isovolumes in the outflow cannula color coded by wall distance to highlight the penetration depth of that region under baseline conditions and the effect of the AP on its clearance.

The bend relief and resulting curvature of the HM3 outflow lead to the formation of a flow separation (Figure 9A), which accounts for the majority of the OBS remaining in the outlet by *V*_*f*_ = 2.0. By increasing mixing during the low-speed phase and then quickly removing mixed blood during the acceleration, the AP improves washout of the bend relief (Figure 9B) and generally reduces OBS concentrations in the outlet (Figures 5C1,C2).

## Discussion

Left ventricular assist device thrombogenicity depends on multiple factors including patient-specific characteristics (24) as well as pump implantation configurations (25, 26), postimplant patient management strategies (27), and the pump internal flow fields (6), which, in turn, affect blood cell and protein residence times and shear stress exposure. Aiming at providing mechanistic insights into the low occurrence of the HM3 in-pump thrombosis and at isolating the contribution of the AP from that of the HM3 internal design, we used CFD simulations coupled with passive scalar advection to assess the effects of the AP on the volume and surface washout of the HM3. Of specific interest was: (i) the clearance of the last 5 to 1% of residual OBS concentration, taken as indicative of regions of prolonged residence times and (ii) the clearance of the pump internal surfaces to assess the effect of the AP in mitigating the risk of thrombus formation and/or deposition inside the pump.

Different measures of scalar washout and residence time have been studied to compare the performances across different pumps or flow conditions. Molteni et al. (28) compared the outlet scalar washout of the CentriMag (Abbott, Chicago, Illinois, USA), HVAD, and the HMII for pump flow rates ranging between 1 and 5 L/min. After normalization for the effect of pump priming volume and flow rate, the time required for the residual OBS concentration at the pump outlet to drop below 50, T50, corresponded to 0.76–0.94 flush volumes for all the pumps and all the flow conditions. Time required to reduce the residual outlet OBS concentration to 5%, T05, showed higher variations, corresponding to 1.8–2.2 flush volumes for the HMII up to 2.6–2.8 in the HVAD and CentriMag. Deriving the outlet scalar washout from our results (Supplementary Material S4), the T50 in the HM3 under the baseline, high-, and low-flow conditions corresponded to 0.9 flush volumes for all the three conditions, while T05 ranged between 1.6 and 1.7. Zhang et al. (29) reported average residence times of 0.12, 0.20, and 0.18 s at the outlet of the CH-ventricular assist device (CH-VAD) (CH Biomedical Incorporation, Suzhou, China), HVAD, and the HM II for a pump flow rate of 4.5 L/min. A similar metric can be derived from the washout plots under constant operating conditions (Supplementary Material S5), yielding average residence times of 0.28, 0.18, and 0.11 s under the low-flow, baseline, and high-flow conditions, respectively. These average residence times correspond to 0.97 flush volumes for all the three conditions. By investigating the performance of the HVAD under different constant operating conditions, Granegger et al. (30) reported that the 90% washout time, defined as the time required to reduce the residual old blood concentration within the pump from 100% down to 10%, was 0.3 s at 5 L/min and 0.6 s at 2.5 L/min, scaling inversely with the flow rate. Mean blood transit times reported by Bourque et al. (11) for the HM3 also followed a perfect inverse-proportional relationship with the pump flow rate, with values of 0.587, 0.219, and 0.118 s at 2.0, 5.4, and 10.0 L/min, respectively. Similarly, in this study, the 95% washout of the HM3, defined as the number of flush volumes required to reduce the residual old blood concentration in the pump from 100 down to 5%, was comparable across tested conditions. Whether the AP was turned on or off, 95% washout was achieved within 1.3–1.4 flush volumes. Similar results were obtained by Boraschi et al. (17) under a different baseline condition, wherein the 95% washout time was 0.29 s at 5 L/min, translating to 1.4 flush volumes, a performance which appears reasonable when compared to the best possible scenario wherein 95% washout would be achieved in exactly 0.95 flush volumes. Altogether, despite the large and rapid changes in both the flow rate and rotor speed imparted by the AP, washout dynamics down to 95% pump washout are only marginally affected when normalizing for the effect of flow rate. Similarly, average residence times were primarily dictated by the flow rate, corresponding to the time to drive approximately one flush volume of blood through the pump. This suggest that, overall, the 95% washout is primarily determined by the pump flow, while the acceleration and deceleration rates of the rotor and flow only have a marginal effect. These further point to a predominant role of the pump internal design and smaller role of the AP at least for the washout of main flow paths.

On the other hand, the AP showed to be of advantage for the 99% washout of the HM3. At baseline, reducing the rOBS concentration down to 1%, i.e., achieving 99% washout, required 2.5 flush volumes, a stark increase compared to the 1.4 flush volumes required for the 95% washout. When accounting for differences in flow rate, 99% washout was faster with the AP than without for all the considered injection times (*V*_*f*_ = 2.1–2.2), suggesting that the AP may help clear harder to wash fluid regions. This is of interest, as fluid regions with prolonged high OBS concentrations pinpoint areas with prolonged residence times, which although the exact thresholds remain elusive and no doubt contributes to increased risk of platelet aggregation and deposition.

Closer investigation of the pump clearance suggests that the AP may be beneficial for the clearance of the near-wall regions in the inlet and outlet as well as of the flow separation downstream of the bend relief. It is not unexpected that curvature—which is needed to connect the pump to the aorta—may lead to flow separation (Figure 9). In the HM3, part of that curvature is already included within the outflow cannula by the bend relief. The AP benefits the clearance of that specific region, increasing mixing during the low-flow phase followed by rapid clearance during the high-speed phase. Whether such effect would also benefit graft washout downstream of the pump remains to be demonstrated. Clearance of the outflow graft, which has until now received little attention, may be a relevant topic to pursue in the future, especially since these grafts are also prone to thrombus formation (31).

In contrast to the inlet and outlet cannulas, washout of the HM3 pump core benefitted only minimally from the AP (Figure 5), pointing to a primary role of the rotor and volute design in the observed volume washout rates despite the large changes in local flow structures imparted by the AP. Specific design features of the HM3 pump core include no central post and large gaps. Consistent with the results of Fang et al. (32) and Wiegmann et al. (18), the absence of central post leads to the formation of a flow stagnation region centrally below the rotor under baseline conditions. This stagnation region was completely eradicated by flow reversal during the low-speed phase of the AP. In the top gap, the competing effect of centrifugal forces and pressure gradients leads to a complex alternation of recirculation regions and the formation of a stagnation point along the upper surface of the rotor. By changing the rotor speed, the AP again favors spatial shift of the stagnation points and mixing, which may theoretically be advantageous for washout. However, the noted stagnation regions are not stable even under constant operating conditions. rOBS concentration centrally under the rotor is already below 1% after 1.4 flush volumes at baseline. Bottom, side, and top secondary flow paths, although cleared at a slower rate than the main flow path, do not display isolated pockets of high rOBS values (Figures 6D2,D3), suggesting adequate washout for all the considered conditions.

Besides OBS washout (or volume washout), the impact of the AP on WSS is of interest, as it directly impacts surface washout. WSS provides an indication of near-wall blood velocity and regions of low WSS (<0.3 or 1 Pa) have been associated with an increased risk for clot deposition (33, 34). Jamiolkowski et al. (35) further reported that wall shear rates above 1,000 s^−1^ [corresponding to WSS of 3 Pa (36) with viscosity used in their study] were related to decreased thrombus stability and more embolization events. Within the stagnation region centrally below the rotor, maximum WSS reached 15.6 Pa at baseline, which is already one order of magnitude higher than reported cutoff values for clot deposition or thrombi destabilization and up to 576 Pa during the AP, over two orders of magnitude higher than reported thresholds. The high WSS value and changing WSS direction observed in the HM3 pump core are, therefore, likely to further contribute to the very low rate of in-pump thrombosis noted in patients with the HM3. However, further studies would be needed to determine whether such high WSS and WSS gradients are required to that end or whether the same objective may be achieved with already lower values. Whether periodic surface clearance, as imparted by the AP, enhances the risk of secondary thromboembolic events caused by the detachment of small thrombi formed on the pump surface also warrants further investigation.

Finally, the above noted benefits of the AP in terms of volume and surface washout also come at the cost of an abrupt increase in fluid shear stresses throughout the pump volume. As discussed by Wiegmann et al. (18) and Boraschi et al. (17), the rotor acceleration and high-speed phase of the AP increase fluid shear stresses and turbulence levels. In both the studies, the stress-exposure profiles of particles flowing through the pump during the AP shifted toward longer exposure time to supraphysiological stresses combined with rapid fluctuations in experienced shear stresses, both of which were experimentally found to contribute to platelet activation (7). The high stresses imparted by the AP may, therefore, contribute to the still relevant rates of strokes in patients implanted with the HM3 (9). Elevated fluid shear stresses and turbulence have also been associated with the degradation of von Willebrand factor (vWF) multimeres (37) that are essential for hemostasis. Clinically, although at a lower extent compared to the HeartMate II, vWF deficiency (38, 39) and bleeding rates (42.9%) (9) remain high with the HM3. A rationale revision of the AP speed modulation sequence for lower shear stress exposures may be, therefore, an interesting avenue to pursue to reduce its intrinsic prothrombotic profile and limit bleeding complications. Of note, lowering shear stress generated by the pump during the AP might reinforce the clinical reliability of new antithrombotic strategies—lower anticoagulation target (40) or aspirin-free regimen (39, 41, 42), which showed improved hemocompatibility-related outcomes in patients with HM3.

This study has limitations. Blood was simplified to an incompressible Newtonian fluid, excluding particles and semisolid components, such as cells, proteins, and lipoproteins. The HM3 geometry was scanned from an explanted pump. Therefore, we cannot exclude small differences to the actual geometry. Furthermore, the HM3 features textured blood-contacting surfaces, which were not taken into account in this study, but have been assumed, also contribute to enhanced antithrombogenic properties (11). Residual cardiac contractility was not simulated, meaning that we neglected the impact of cardiac pulsatility on internal pump hemodynamics, which is expected to influence washout time because of its influence on the mean pump flow. However, as changes imparted by the residual (low) cardiac contractility of patients with end-stage heart failure are smoother and of lower amplitude than those imparted by sudden changes characteristics of the AP, we expect the normalized behavior to be well captured by the conditions investigated here. The HM3 only allows to increase/decrease the pump rotational speed by a 100-rpm step and an “unphysical” baseline rotational speed of 5,650 rpm was used. This choice was dictated by the numerical problem, specifically by the need to target clinically relevant flow conditions. In detail, according to the patient characteristics of our lumped parameter model of the cardiovascular system, this value allowed us to achieve a mean pump flow rate of about 5 L/min with the AP. On the other hand, results of the simulations where the high- and low-flow conditions were imposed (Supplementary Materials S3–S5) suggest that this 50-rpm difference is unlikely to significantly affect the results. Finally, exact biological thresholds (e.g., WSS needed to prevent deposits on pump surfaces) are unknown. The forces required for the removal of deposits on blood-contacting surfaces depend on the thrombus stability, size, and shape as well as on the surface properties. Further fundamental studies are warranted to evaluate the influence of WSS on deposit removal and blood damage.

## Conclusion

In summary, the AP did not affect the 95% washout, suggesting the dominant role of pump flow rate. The AP helped in clearing regions with prolonged exposure times, particularly in the near-wall regions and bend relief. In contrast and as highlighted by earlier studies, the AP leads to an abrupt increase in turbulence and shear stress levels, which have been associated with higher platelet activation levels and blood damage. Altogether, these results provide a mechanistic rationale as to why the HM3 is associated with negligible rate of in-pump thrombosis, but still relevant rate of stroke. Further understanding of the biological effects of turbulence as well as of the WSS required to prevent thrombus deposition may help to establish the costs and benefits of rotor accelerations such as those imposed during the AP and further optimize rotor speed modulation sequences. From this perspective, our numerical platform represents a valuable tool for the fine-tuning of rotor speed modulation sequences of current and future generations of LVADs.

## Data Availability Statement

The raw data supporting the conclusions of this article will be made available by the authors, without undue reservation.

## Author Contributions

PF and AB formalized the simulation methodology. PF conducted the simulations and performed the data analysis. MS, SB, and AR guided the data interpretation. PF, JD, DZ, and VK were involved in funding acquisition. FC and DZ conceived the study and DZ, JD, and VK directed the research. All the authors contributed to the manuscript and have approved the final version of the manuscript.

## Funding

The authors gratefully acknowledge the financial support from the University of Zürich by contributing to the open access publication fees, the Shenzhen Science and Technology Innovation Foundation (Grant No. JCYJ20160427184134564 to JD), the National Natural Science Foundation of China (Grant No. U2141210 to JD), the China Scholarship Council (No. 202006120445 to PF), the Swiss National Science Foundation through the NCCR Kidney. CH (to VK), and the Stavros Niarchos Foundation (to VK). This study is a part of the Zurich Heart project under the umbrella of University Medicine Zurich.

## Conflict of Interest

The authors declare that the research was conducted in the absence of any commercial or financial relationships that could be construed as a potential conflict of interest.

## Publisher's Note

All claims expressed in this article are solely those of the authors and do not necessarily represent those of their affiliated organizations, or those of the publisher, the editors and the reviewers. Any product that may be evaluated in this article, or claim that may be made by its manufacturer, is not guaranteed or endorsed by the publisher.
