# Supplementary material for: Insights Into the Low Rate of In-Pump Thrombosis With the HeartMate 3: Does the Artificial Pulse Improve Washout?
Source: Front Cardiovasc Med. 2022 Mar 11;9:775780. doi: 10.3389/fcvm.2022.775780 (PMC8962620; doi:10.3389/fcvm.2022.775780)
Supplement: Supplementary file 1 [file Data_Sheet_1.PDF]

Supplementary Material

Insights into the low rate of in-pump thrombosis with the HeartMate 3: Does the artificial pulse improve washout?

Peng Fang, Jianjun Du, Andrea Boraschi, Silvia Bozzi, Alberto Redaelli, Marianne Schmid Daners, Vartan Kurtcuoglu, Filippo Consolo, Diane de Zélicourt

S1. Wall shear stress monitoring points

To evaluate the effect of the artificial pulse (AP) on surface washout, we monitored instantaneous wall shear stress (WSS) at selected locations spanning along the top and bottom of the rotor casing (Figure S1).

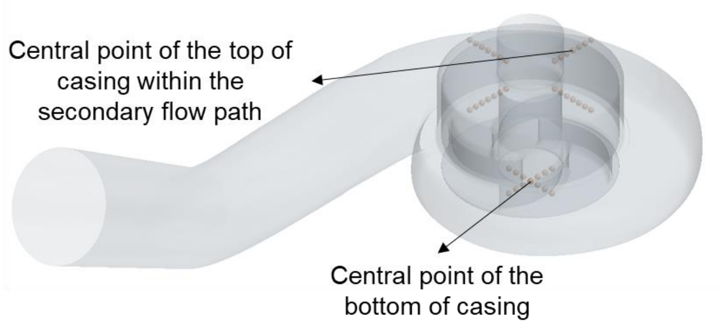

Supplementary Figure 1. Selected locations along the top and bottom of the casing for instantaneous WSS analysis

S2. Agreement between OBSr and OBSref

As illustrated in Figure S2, the residual OBS concentrations in the HM3 closely follow the reference curve, rOBSref, down to a residual concentration of about 30%. For Baseline and AP1-4, the difference between rOBS and rOBSref remains below 0.001% until rOBS=50% and below 0.1% for AP5 between 50 and 40%. The difference further remains below 1% between until rOBS = 30% for Baseline and AP1-3. For AP4, the deviation increases with the start of the AP deceleration phase. For AP5, early onset of the AP leads to part of the old blood to flow back through the inlet, not taken into account in OBSref and leading to a slightly larger deviation from the AP deceleration phase onwards. In all cases difference remains below 2% until rOBS=30%.

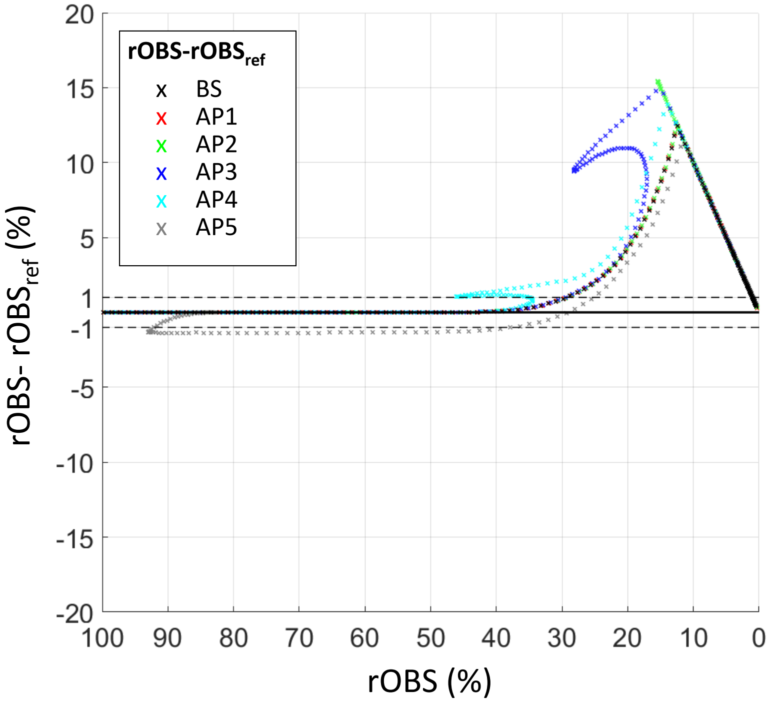

Supplementary Figure 2. Difference between the residual OBS concentration as obtained from the simulations, rOBS, and the reference residual OBS concentration, $rOBS_{ref}$, at Baseline and for the five initialization time points considered with the artificial pulse (AP1-5). Simulation data are downsampled by a factor of 40 to ease visualization.

S3. Effect of high and low flow on OBS washout

In order to shed further light on the role of different rotor speeds and pump flow rates on old blood washout, we conducted two additional simulations: one at high flow and high speed (8.5 L/min, 7600 rpm) and one at low speed (3.5 L/min, 3600 rpm). The decline of the old blood residual fraction during the AP and under the different constant flow conditions (baseline, high and low flow conditions) are provided in Supplementary Figures 3 to 5.

Irrespective of the flow rates or rotor speeds as well as of the static or dynamic nature of the boundary conditions, washout dynamics down to 95% pump washout (i.e. down to an OBS residual fraction of 5%) are only marginally affected when normalizing for the effect of flow rate (Supplementary Figures 3B). However, we do see a benefit of both the AP and the high flow condition when going do 99% washout. Consistent with the AP data, washout of the pump core was only marginally affected by the different conditions after normalizing for the flow rate – suggesting that the latter is the primary driver of core pump washout irrespective of the rotor sequence or flow acceleration/deceleration rates. Regions that most benefitted from the high flow condition were the near wall region of the inlet and outlet and the flow separation downstream of the bend-relief. It is interesting to note that both high and low flow conditions benefitted clearance of the bend relief: by reducing the extent of the flow separation at lower flow rates, and by increasing secondary flow structures and swirling in the outflow cannula, which in turn improved clearance of the flow separation under high flow conditions.

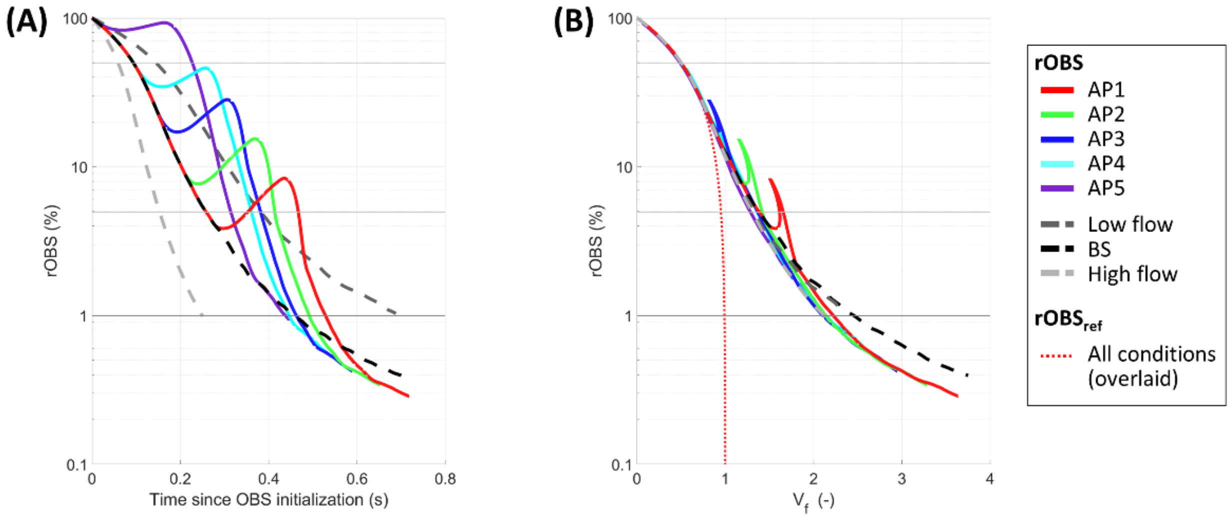

Supplementary Figure 3: Comparison of the old blood scalar washout during the artificial pulse against different constant flow and rotational speed conditions. A) Decline of the residual fraction of old blood within the pump as a function of time. B) Decline of the residual fraction of old blood within the pump as a function of the number of flush volumes run through the pump. rOBS: residual fraction of old blood in the whole pump. AP1 to AP5: Artificial pulse for five different initialization times. BS: Baseline conditions (5.4 L/min, 5650 rpm). Low flow: (3.5 L/min, 3600 rpm). High flow (8.6 L/min, 7600 rpm).

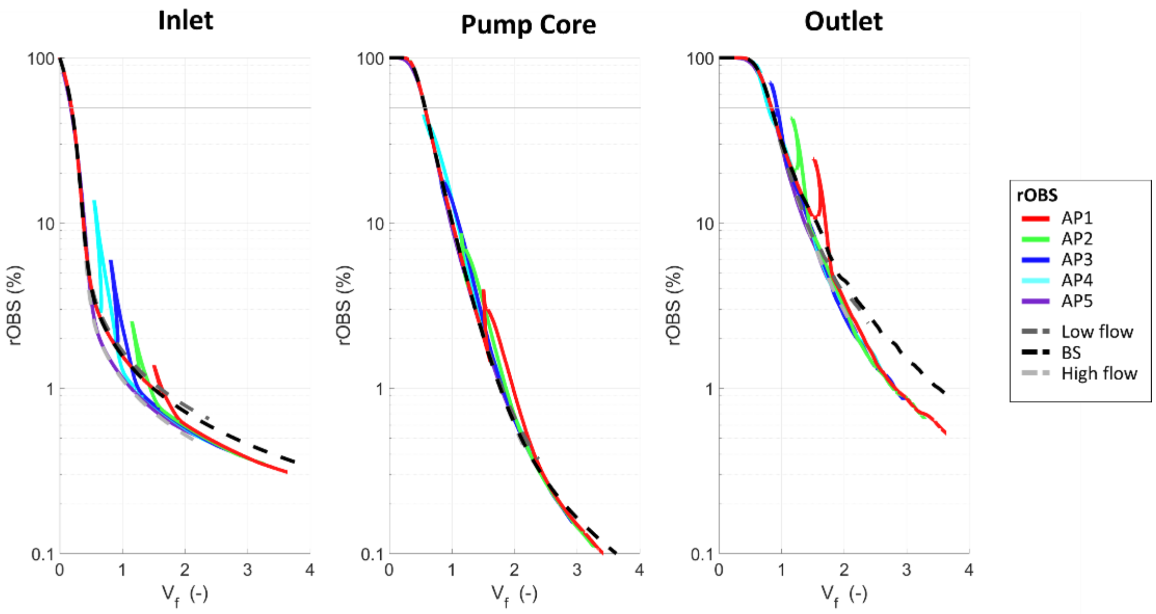

Supplementary Figure 4: Comparison of the old blood scalar washout in the different pump parts during the artificial pulse and under different constant flow and rotational speed conditions. rOBS: residual fraction of old blood in the corresponding pump part. AP1 to AP5: Artificial pulse for five different initialization times. BS: Baseline conditions (5.4 L/min, 5650 rpm). Low flow: (3.5 L/min, 3600 rpm). High flow (8.6 L/min, 7600 rpm).

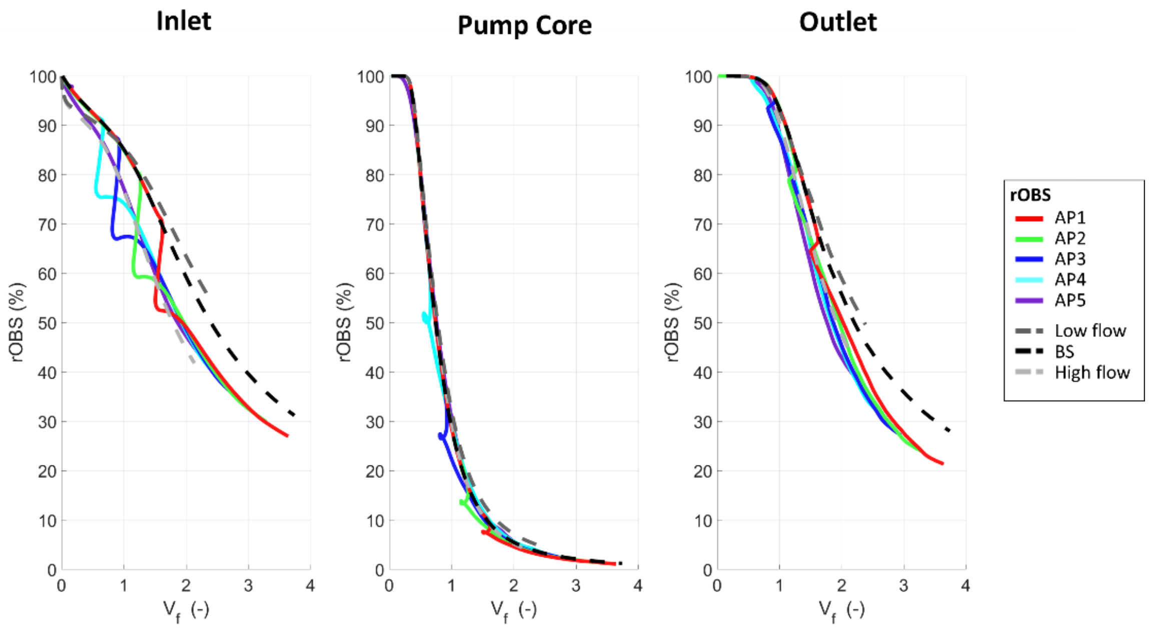

Supplementary Figure 5: Comparison of the old blood scalar washout in the near wall region of the different pump parts during the artificial pulse and under different constant flow and rotational speed conditions. rOBS: residual fraction of old blood in the corresponding pump part. AP1 to AP5: Artificial pulse for five different initialization times. BS: Baseline conditions (5.4 L/min, 5650 rpm). Low flow: (3.5 L/min, 3600 rpm). High flow (8.6 L/min, 7600 rpm).

S4. Outlet washout times calculations

Molteni et al.(1) compared the outlet washout performance of three different devices, namely the CentriMag (Abbott, Chicago, IL, USA), HeartWare HVAD (Medtronic, Minneapolis, MN, USA) and HeartMate II (Abbott, Chicago, IL, USA). In that paper, outlet washout is defined as the mean old blood scalar concentration on the outlet cross-section of the pump, $[OBS]_{outlet}$, and the characteristic washout times, T50 and T05, defined as the time required for $[OBS]_{outlet}$ to reach 50% and 5% of its initial value, respectively. A similar metric can be derived from our data using conservation principles.

Considering the whole pump volume, the rate of change in residual OBS concentration is given by:

$$\frac{d(rOBS)}{dt} = \frac{1}{V_{pump}}(Q \cdot rOBS_{inlet} - Q \cdot rOBS_{outlet})$$

where rOBS is the mean residual OBS concentration inside the pump, Vpump is the pump volume, Q the pump flow rate and $rOBS_{inlet}$ and $rOBS_{outlet}$ the mean residual OBS concentration at the inlet and outlet cross-sections, respectively.

Under our constant operating conditions, $rOBS_{inlet}$ is equal to zero at all times and $rOBS_{outlet}$ can be calculated as:

$$rOBS_{outlet} = \frac{V_{pump}}{Q} \cdot \frac{d(rOBS)}{dt}$$

This assumption does not hold for the artificial pulse, as reverse flow may lead to a transient outflux of old blood scalar from the pump back into the ventricle (i.e. $Q \cdot rOBS_{inlet}$ may not be equal to zero at all times), preventing the reconstruction of $rOBS_{outlet}$ from the rOBS curves alone.

The reconstructed $rOBS_{outlet}$ curves for the baseline, low and high flow scenarios are illustrated in Supplementary Figure 6 and the corresponding T05 and T50 listed in Supplementary Table 1.

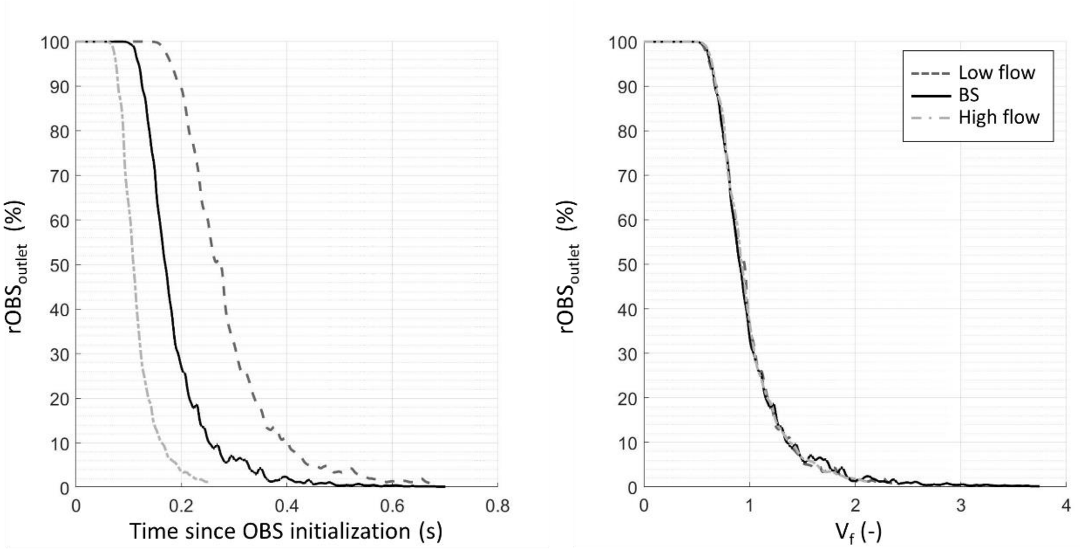

Supplementary Figure 6. Comparison of the outlet washout in the HM3 under baseline, high and low flow condition.

Supplementary Table 1: Time required to reach 50% and 5% of the initial OBS concentration at the outlet under different constant operating conditions.

Operating condition	Flow rate (L/min)	T50		T05	
		Time (s)	V_f (-)	Time (s)	V_f (-)
Low flow	3.50	0.27	0.92	0.45	1.57
Baseline	5.42	0.17	0.91	0.32	1.70
High flow	8.60	0.11	0.92	0.19	1.65

S5. Mean residence time calculation

Under constant operating conditions, the mean residence time of the blood travelling through the pump can be derived from the old blood scalar washout curves as detailed below.

- Let us consider two consecutive washout characterization separated by a time interval Δt (OBSa and OBSb in Supplementary Figure 7.A). OBSa corresponds to the blood that entered, and did not yet exit the pump, up until the time point t_a and is then replaced by new blood after t_a. Similarly OBSb corresponds to the blood that entered and did not yet exit the pump up until time point $t_b = t_a + \Delta t$.
- We can define ΔOBS = OBSb-OBSa, which describes the blood that entered the pump between the times t_a and t_b. The temporal evolution of the residual fraction of ΔOBS in the pump (i.e. ΔOBS normalized by its value in t_b) is illustrated in Supplementary Figure 7.B. ΔOBS initially remains constant as blood travels from the inlet towards the outlet. It starts declining as the fastest blood elements reach the outlet.
- The temporal derivative of ΔOBS describes the rate at which blood exists the pump at every time point. The probability density function of blood reaching the outlet at a given time is given by:

$$\Pr(t) = \frac{\frac{\partial \Delta OBS}{\partial t}(t)}{\int \frac{\partial \Delta OBS}{\partial t}(\tau)\partial\tau}$$

And the mean residence time, mRT, is given by:

$$mRT = \int \tau \cdot \Pr(\tau)\,\partial\tau$$

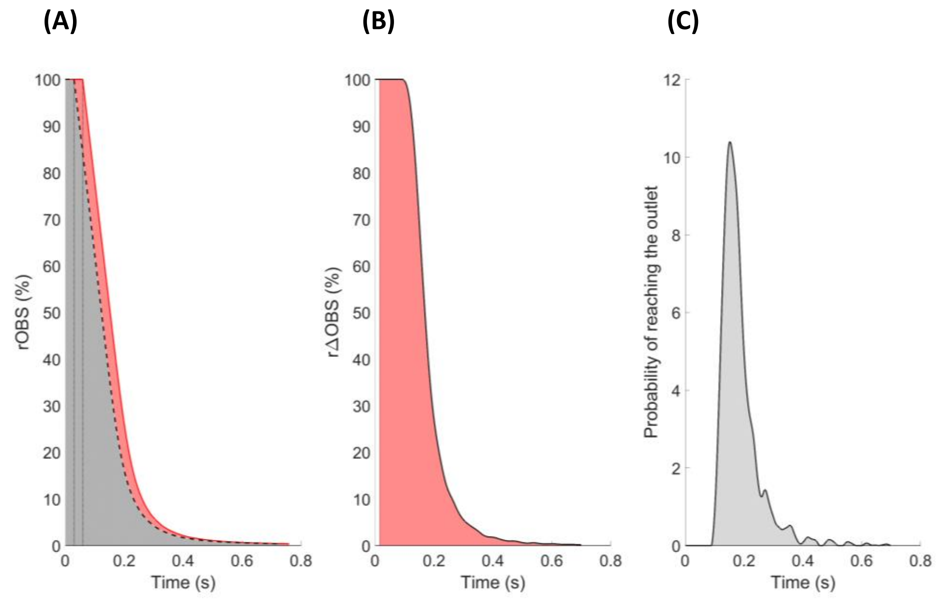

Supplementary Figure 7. Mean residence time derivation.

Old blood scalar washout under constant operating conditions can be considered self-similar irrespective of the initialization time point and we can assume that OBSb follows the same time course as OBSa with a time shift Δt, such that ΔOBS may be defined from a single washout curve as: ΔOBS = OBS(t)- OBS(t+ Δt). This assumption does not hold under dynamic operating conditions.

The assumption of self-similarity was confirmed for the baseline scenario, comparing 5 different OBS washout curves (Supplementary Figure 8). Maximum deviation between individual curves and the mean of the 5 curves were below 0.3% for rOBS ranging between 100 and 0.5% (Supplementary Figure 9). Supplementary Table 2 summarizes the mean residence times derived from the simulated OBS washout for the three constant operating conditions, applying the approach detailed above.

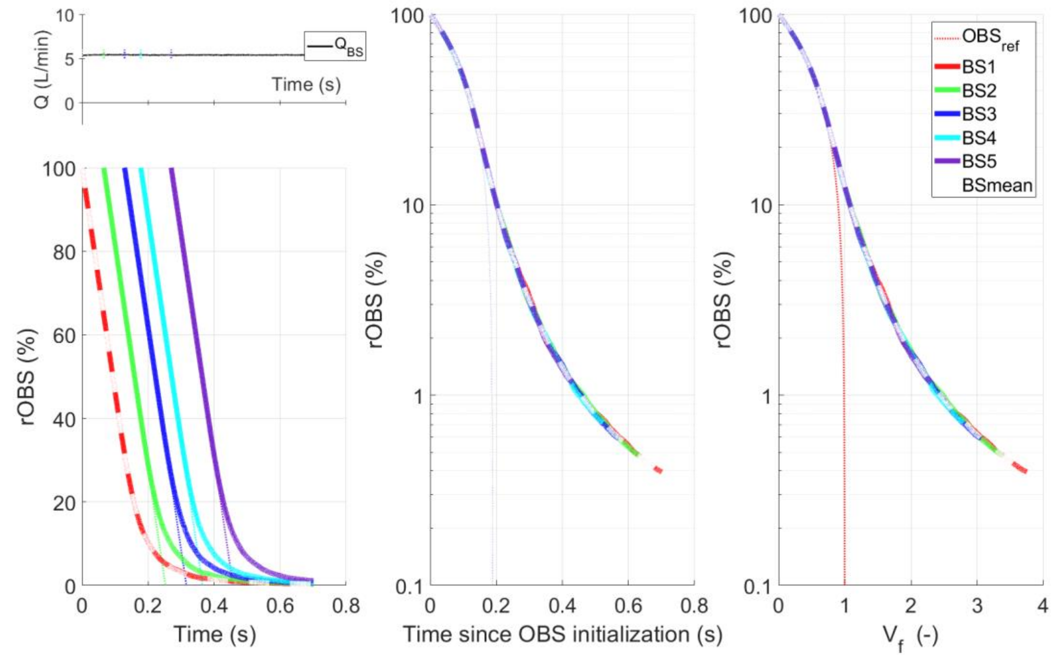

Supplementary Figure 8. Sensitivity of the OBS washout on its initialization time under baseline conditions.

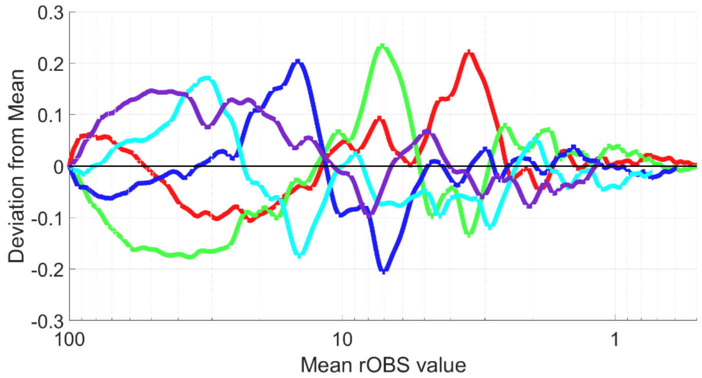

Supplementary Figure 9. Deviation between individual rOBS curves and the mean of the five curves as a function of the mean rOBS value.

Supplementary Table 2: Mean residence times under different constant operating conditions.

Operating condition	Mean residence time		Flow rate (L/min)
	Time (s)	V_f (-)	
Baseline	0.18	0.97	5.42
Low flow	0.28	0.97	3.50
High flow	0.11	0.96	8.60

References

1. Molteni A, Masri ZP, Low KW, Yousef HN, Sienz J, Fraser KH. Experimental measurement and numerical modelling of dye washout for investigation of blood residence time in ventricular assist devices. *Int J Artif Organs* (2018) **41**:201–212. doi:10.1177/0391398817752877
